# Public health round-up

**DOI:** 10.2471/BLT.22.010422

**Published:** 2022-04-01

**Ukraine**

A girl stands before a devastated apartment building in Kyiv, Ukraine where damage to civilian infrastructure resulting from shelling and bombardment has left hundreds of thousands of people without shelter. As of 14 March, some 18 million people had been affected by the war in Ukraine, including 3 million refugees who had fled to neighbouring European countries.

**Figure:**
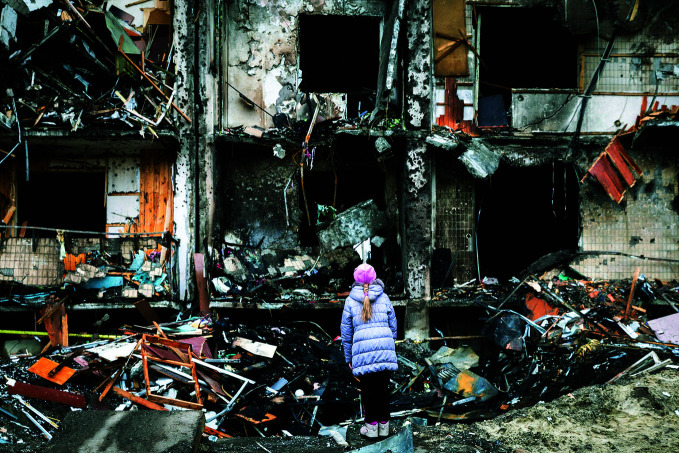
UNICEF/Anton Skyba

## Ukraine crisis

The Russian Federation launched a military offensive against Ukraine, triggering a humanitarian emergency affecting Ukraine and surrounding countries. The offensive began on 24 February and has since escalated with multiple attacks on Ukrainian ports, infrastructure – including health infrastructure – and cities.

As of 14 March, an estimated 18 million people in Ukraine had been affected, including 6.7 million people who had been internally displaced. Nearly 3 million people had fled the country.

The World Health Organization (WHO) is coordinating with partners to ensure that neighbouring countries have the infrastructure and expertise required to meet the urgent needs of refugees.

WHO and partners are also working to support Ukraine’s health system by shipping life-saving equipment and medical supplies that include oxygen generators, generators to maintain electrical supply in affected health facilities, defibrillators, monitors, anaesthesia drugs, rehydration salts, gauzes, bandages and blood transfusion kits. All supplies are being distributed in close coordination with the Ukraine Ministry of Health, based on WHO critical needs assessments, public health risk, service assessments and logistic capacity.

As mandated by World Health Assembly Resolution 65.20 which was adopted in 2012, WHO is also closely monitoring attacks on Ukrainian health care. As of 12 March, the Organization had verified a total of 31 such attacks between 24 February and 11 March 2022, resulting in 12 deaths and 34 injuries. Eight of the injured and two of those killed were health workers.

In a joint statement issued on 13 March, WHO, the United Nations Children’s Fund (UNICEF) and the United Nations Population Fund called for an immediate cessation of all attacks on health care in Ukraine. "To attack the most vulnerable – babies, children, pregnant women, and those already suffering from illness and disease, and health workers risking their own lives to save lives – is an act of unconscionable cruelty,” the statement read.

https://bit.ly/35VGwIT

https://bit.ly/3CEFXz2

https://bit.ly/3tMP7FK

## Wild poliovirus in Malawi

The presence of type 1 wild poliovirus (WPV1) was confirmed in a three-year-old girl suffering from paralysis in Tsabango, Lilongwe, Malawi. The girl experienced onset of paralysis on 19 November 2021, and stool specimens were collected for testing on 26 and 27 November. Sequencing of the virus conducted in February by the National Institute for Communicable Diseases in South Africa and the United States of America’s (USA) Centers for Disease Control and Prevention confirmed this case as WPV1.

The Global Polio Eradication Initiative (GPEI) partners, including WHO, are supporting health authorities in Malawi as they assess the situation and begin urgent immunization activities in the subregion to mitigate the risk of spread. Surveillance measures are also being expanded in Malawi and neighbouring countries to detect any other potential undetected transmission.

A GPEI Rapid Response Team was sent to Malawi to support coordination, surveillance, data management, communications and operations. Partner organizations also sent teams to support emergency operations and innovative vaccination campaign solutions.

In a 17 February announcement, GPEI stated that the virus is genetically linked to WPV1 that was detected in Pakistan’s Sindh province in October 2019.

A meeting of the Emergency Committee under the International Health Regulations (2005) on the international spread of poliovirus was convened on 28 February. The committee reviewed the data and underlined several concerns including an unknown chain of transmission from Pakistan to Africa.

The committee unanimously agreed that the risk of international spread of poliovirus remains a Public Health Emergency of International Concern and recommended the extension of temporary recommendations for a further three months.

https://bit.ly/3KLhlHG

https://bit.ly/3hDseih

## Germany pledges “fair share” to ACT-Accelerator

Germany pledged to meet its “fair share” of the Access to COVID-19 Tools (ACT) Accelerator’s 2021/22 budget, with a contribution of US$ 1.22 billion to support the partnership’s vital work on access to COVID-19 treatments, tests, vaccines and personal protective equipment. A detailed breakdown of the funding allocation to the ACT-Accelerator’s constituent agencies will be confirmed at a later stage.

Announced on 1 March, the pledge followed the 9 February announcement of a new “fair share framework” by the ACT-Accelerator resource mobilization group and an appeal by leaders for governments of higher-income countries to meet the US$ 16 billion ACT-Accelerator funding gap and US$ 6.8 billion in-country delivery costs to support efforts to end the pandemic.

https://bit.ly/3HBWpkE

## Biomanufacturing training hub in Republic of Korea

WHO, the Republic of Korea and the WHO Academy announced the establishment of a global biomanufacturing training hub that will serve all low- and middle-income countries wishing to produce biologicals, such as vaccines, insulin, monoclonal antibodies and cancer treatments. Announced on 23 February, the move comes after the successful establishment of a global mRNA vaccine technology transfer hub in South Africa.

“One of the key barriers to successful technology transfer in low- and middle-income countries is the lack of a skilled workforce and weak regulatory systems,” said WHO Director-General Tedros Adhanom Ghebreyesus. “Building those skills will ensure that they can manufacture the health products they need.”

https://bit.ly/35ruC9h

## New COVID-19 technologies for C-TAP

The United States National Institutes of Health announced that it will be offering several innovative therapeutics, vaccines and diagnostic methods to the COVID-19 Technology Access Pool (C-TAP) for potential licensing through the Medicines Patent Pool.

The announcement was made on 3 March at the USA’s COVID-19 Dialogue with Ministers of Health meeting in the presence of WHO Director-General Tedros Adhanom Ghebreyesus, and The National Institute of Allergy and Infectious Diseases Director, Dr Anthony Fauci. An announcement regarding which technologies are to be included will be made once details have been worked out.

Launched in 2020 by the WHO Director-General and the President of Costa Rica, and supported by 43 Member States, C-TAP aims to facilitate timely, equitable and affordable access to COVID-19 health products by boosting their production and supply through open, non-exclusive licensing agreements.

https://bit.ly/35yFUsm

**Cover photo**

A refugee family fleeing Ukraine enters Romania at the Isaccea border crossing.

**Figure:**
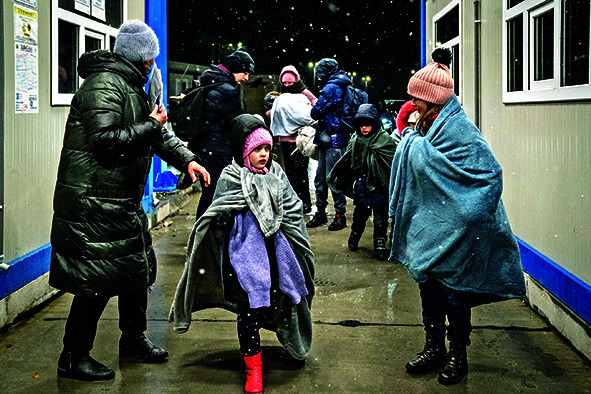
UNICEF/ UN0599555/Moldovan

## Reducing antimicrobial waste

The Global Leaders Group on Antimicrobial Resistance called on all countries to reduce the amount of antimicrobial waste entering the environment. In a 2 March statement the group, which includes heads of state, government ministers, and leaders from private sector and civil society, appealed for countries to develop and implement regulations and standards to better monitor and control the distribution and release of antimicrobials and drug-resistant organisms into the environment.

Specific recommendations included: the development of national antimicrobial manufacturing pollution standards; the enforcement of laws designed to reduce or eliminate antimicrobial use that is not under the guidance of a trained health-care provider in the human and animal health sector; and the implementation of standards to treat and manage discharge from food-animal farms, aquaculture farms and crop fields.

https://bit.ly/35oC0SQ

## Aggressive formula milk marketing

Roughly one in two parents are targeted with marketing from formula milk companies, much of which is in breach of international standards on infant feeding practices.

This is one of the main conclusions of a new WHO/UNICEF report based on interviews with parents, pregnant women and health workers in eight countries that was published on 22 February. *How the marketing of formula milk influences our decisions on infant feeding* uncovers systematic and unethical marketing strategies that range from invasive online targeting and sponsored “advice” networks to promotions and free gifts. Formula milk companies also seek to influence health worker training and practice.

https://bit.ly/3hyc4GT

**Looking ahead**

24–30 April, World Immunization Week. https://bit.ly/3HLbEYq

3–5 May, Geneva Health Forum. https://bit.ly/3HJ5wQt

22–28 May, Seventy-fifth World Health Assembly. https://bit.ly/3IK55GL

